# Heart-Rate-Corrected QT Interval Response to Ramosetron during Robot-Assisted Laparoscopic Prostatectomy: A Randomized Trial

**DOI:** 10.3390/jpm12050811

**Published:** 2022-05-18

**Authors:** Bora Lee, So Yeon Kim, Seung Hyun Kim, Hyukjin Yang, Jeong Hyun Jin, Seung Ho Choi

**Affiliations:** Department of Anesthesiology and Pain Medicine, Severance Hospital, Anesthesia and Pain Research Institute, Yonsei University College of Medicine, 50-1 Yonsei-ro, Seodaemun-gu, Seoul 03722, Korea; dreamkaist@yuhs.ac (B.L.); kimsy326@yuhs.ac (S.Y.K.); anesshkim@yuhs.ac (S.H.K.); medjumper@yuhs.ac (H.Y.); joann94@yuhs.ac (J.H.J.)

**Keywords:** QTc prolongation, ramosetron, robot-assisted laparoscopic radical prostatectomy, Trendelenburg

## Abstract

Ramosetron, often used to prevent postoperative nausea and vomiting, might cause heart-rate-corrected (QTc) interval prolongation, as might robot-assisted laparoscopic prostatectomy (RALP), which requires a steep Trendelenburg position and CO_2_ pneumoperitoneum. This study aimed to determine how ramosetron administration affects the QTc interval in patients treated with RALP. Fifty-six subjects were randomly assigned to ramosetron (n = 28) or control (n = 28) groups. The ramosetron group received 0.3 mg of ramosetron after anesthetic induction, whereas the control group received normal saline. The QTc interval was measured before and after induction; after 5, 30, and 60 min of being placed in the Trendelenburg position; immediately after being returned to a supine position; and at the end of surgery. Linear mixed models were used to compare QT intervals between groups. QTc intervals did not differ significantly between groups over time (P_group×time_ = 0.111). However, they increased significantly in both groups after placement in the Trendelenburg position compared with before induction (P_time_ < 0.001). This increase in QTc continued until the end of surgery in both groups. Based on these findings, ramosetron can be safely administered for the prevention of postoperative nausea and vomiting among patients undergoing RALP.

## 1. Introduction

Antagonists for the 5-hydroxytryptamine 3 (5-HT_3_) receptor are commonly administered for the prevention of postoperative nausea and vomiting [1]. Compared with other 5-HT_3_ receptor antagonists, the recently introduced 5-HT_3_ receptor antagonist, ramosetron, has a prolonged activity duration and an increased binding affinity [2]. However, 5-HT_3_ receptor antagonists have been associated with a heart rate (HR)-corrected prolongation of the QT interval (QTc) [3,4]. QTc interval prolongation has been linked to an enhanced risk of polymorphous ventricular arrhythmias, such as Torsades de Pointes (TdP), which can lead to fatal ventricular fibrillation, cardiovascular mortality, or death [5,6].

Robot-assisted laparoscopic prostatectomy (RALP) is associated with reductions in blood loss, transfusions, hospitalization durations, and recurrence compared with other surgical cancer treatment methods [7,8]. RALP is performed with the patient in a steep Trendelenburg position, combined with CO_2_ pneumoperitoneum, which activates the sympathetic nervous system and affects cardiac repolarization [9,10]. The QTc interval and the interval between the peak of the T wave (Tpeak) and the end of the T wave (Tend), also known as the Tp-e interval, are used as ventricular repolarization indices [6].

Ramosetron is a commonly used 5-HT_3_ receptor antagonist; therefore, it was necessary to investigate whether it can be safely used in surgery that can increase the QTc interval. Previous research suggested that the administration of 0.3 mg of ramosetron during general anesthesia had no effect on the QTc interval [11]. In contrast, another study reported that ramosetron administration prolonged the QTc interval relative to that in the control group [4]. Prolongation of the QTc interval has likewise been reported among patients undergoing RALP [10]. RALP can prolong the QTc interval; however, whether ramosetron affects the QTc interval during RALP has not yet been explored. Therefore, this study aimed to investigate the effects of ramosetron administration on the QTc and Tp-e intervals among subjects undergoing RALP.

## 2. Materials and Methods

### 2.1. Subjects

The study protocol was approved by the Severance Hospital Institutional Review Board, Seoul, South Korea, on 17 July 2017 (No. 4-2017-0487) and registered at ClinicalTrials.gov (No. NCT03232125; 27 July 2017). This study was performed in accordance with the Declaration of Helsinki regarding human rights. Written informed consent was obtained from all enrolled subjects prior to randomization. This single-center study enrolled 56 subjects requiring RALP between December 2017 and June 2020. The exclusion criteria were as follows: any preoperative electrocardiography (ECG) abnormalities, such as a QTc interval > 500 ms; an abnormal ventricular conduction; other arrhythmias; unstable angina; the use of antiarrhythmic agents; use of any medications reported to prolong the QTc interval; or irregular preoperative serum electrolyte levels.

### 2.2. Randomization and Interventions

Enrolled subjects were randomly assigned to either the ramosetron group (n = 28) or the control group (n = 28). Randomization was performed using a computer-generated randomization table (obtained from http://www.random.org, accessed on 13 December 2017). Randomization and group assignments were performed by an anesthesiologist who did not participate in data collection. The ramosetron group was intravenously administered 0.3 mg of ramosetron 15 min after intubation, whereas an equal volume of normal saline was administered to the control group 15 min after intubation. After data collection, ramosetron (0.3 mg) was administered to the control group at the end of the surgical procedure. An anesthesiologist, who did not participate in the data collection process, prepared both the ramosetron and saline solutions. All participants, including the surgeons, study participants, and primary investigator, were blinded to the treatment.

### 2.3. Anesthesia

Propofol at 2 mg/kg, rocuronium at 1 mg/kg, and remifentanil at 0.5–1 µg/kg were used to induce anesthesia. A radial artery catheter and peripheral venous line were inserted following intubation. Sevoflurane (0.8–1 × the age-adjusted minimal alveolar concentration in 50% O_2_/air) and remifentanil (0.05–0.15 µg/kg/min) were used to maintain anesthesia. A SedLine^®^ electroencephalograph sensor (Masimo Corp., Irvine, CA, USA) was used to monitor the Patient State Index. Pulse oximetry, ECG, invasive arterial blood pressure, oropharyngeal temperature, end-tidal carbon dioxide (EtCO_2_) concentration, and end-tidal sevoflurane concentration (EtSevo) were monitored. RALP was performed in the 30° Trendelenburg position with CO_2_ pneumoperitoneum (intra-abdominal pressure, 12 mmHg).

### 2.4. Data Collection and Outcome Assessments

The primary outcome was a change in QTc intervals associated with ramosetron administration during RALP. Secondary outcomes were changes in the Tp-e interval and the Tp-e/QT ratio. Continuous ECG recordings were collected using LabChart software (Pro version 7; AD Instruments, Colorado Springs, CO, USA) and a data-acquisition system (PowerLab; AD Instruments). Serial QT and Tp-e intervals were averaged from the values acquired for four successive beats taken at the following time points: before induction; 10 min after intubation; after 5, 30, and 60 min of being placed in a steep Trendelenburg position with CO_2_ pneumoperitoneum; immediately after being placed in the supine position, with CO_2_ desufflation (T-off); and at the end of surgery (surgery end). HR correction for the QTc interval was performed using Bazett’s (QTcB) and Fridericia’s (QTcF) formulae [12]. At each time point, hemodynamic parameters, such as HR and mean arterial pressure (MAP), were also recorded.

### 2.5. Statistical Analysis

We calculated the required sample size according to the primary outcome of QTc prolongation, and a QTc prolongation > 20 ms between the ramosetron and control groups was defined as clinically relevant [6,10]. A required sample size of 25 participants was determined for each group, based on a power of 80% and a significance level of 5%. Considering a potential 10% dropout rate, 28 subjects were enrolled in each group.

Data regarding demographics and intraoperative variables are presented as the number of subjects (%), mean ± standard deviation, or median (interquartile range). Parametricity was confirmed by the Shapiro–Wilk and Kolmogorov–Smirnov tests. Continuous parametric variables were compared using an independent-samples t-test. Comparisons of non-parametric data were performed using the Mann–Whitney U test. Categorical variables were compared using the χ^2^ or Fisher’s exact test. Linear mixed models using random and fixed between-group effects were used to analyze repeated measurements of QT intervals, MAP, HR, EtCO_2_, EtSevo, Tp-e interval, and Tp-e/QT ratio. Intergroup comparisons of changes in variables over time were performed using a group-by-time interaction, and compound symmetry was used to correlate repeated measures. Bonferroni correction was applied to post hoc analyses to identify statistical differences at each time point compared with before induction. A *p*-value of <0.05 was considered statistically significant. All statistical analyses were performed using SPSS v25.0 (IBM Corp., Armonk, NY, USA), R version 3.5.1 (R Foundation for Statistical Computing, Vienna, Austria), and SAS v9.4 (SAS Inc., Cary, NC, USA).

## 3. Results

**Figure 1:**
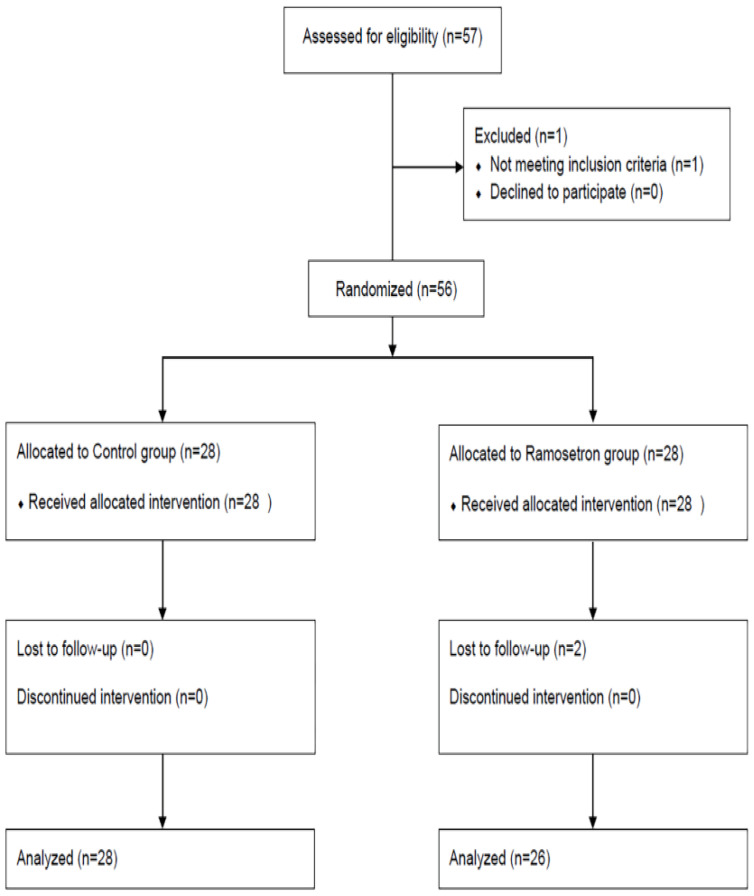
Flow diagram of the study subjects.

A total of 57 subjects were evaluated for study eligibility between December 2017 and June 2020, among whom 56 were enrolled and randomly assigned to the ramosetron (n = 28) or control group (n = 28). The failure to obtain QTc interval data due to T-wave flattening resulted in the exclusion of two subjects in the ramosetron group (Figure 1).

No significant differences were observed among patient characteristics or intraoperative variables between the two groups (Table 1).

**Figure 2:**
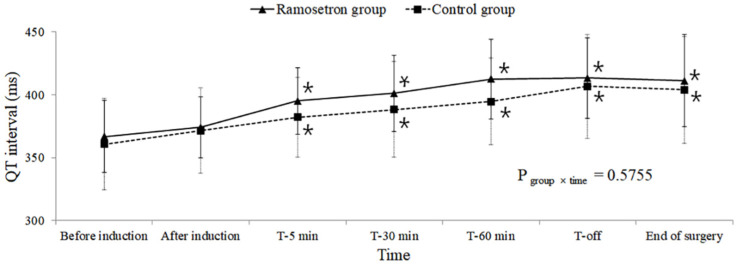
Changes in QT interval during robot-assisted laparoscopic prostatectomy. Values are presented as mean ± standard deviation. T-5 min, 30 min, 60 min: 5, 30, and 60 min after assuming the steep Trendelenburg position with CO_2_ pneumoperitoneum; T-off: Immediately after assuming a supine position with CO_2_ desufflation. * Bonferroni-corrected *p* < 0.05 compared with the value before induction within each group.

**Figure 3:**
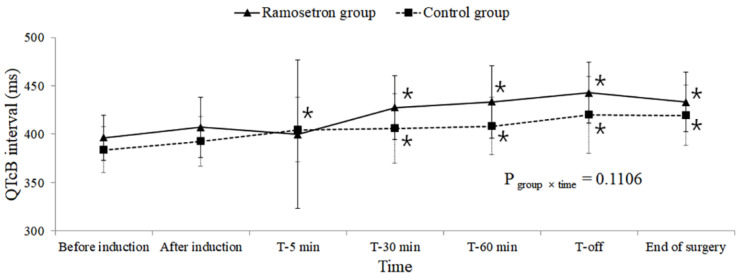
Changes in QTcB during robot-assisted laparoscopic prostatectomy. Values are presented as mean ± standard deviation. QTcB: Heart-rate-corrected QT intervals calculated by Bazett’s formula; T-5 min, 30 min, 60 min: 5, 30, and 60 min after assuming the steep Trendelenburg position with CO_2_ pneumoperitoneum; T-off: Immediately after assuming a supine position with CO_2_ desufflation. * Bonferroni-corrected *p* < 0.05 compared with the value before induction within each group.

**Figure 4:**
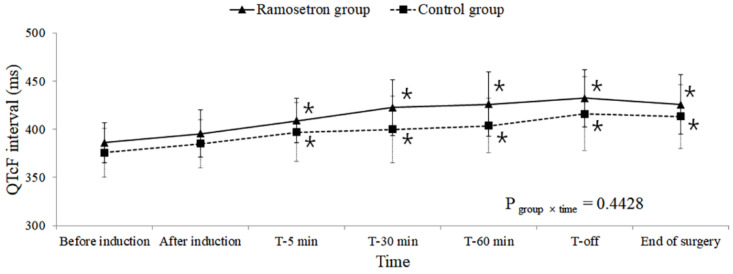
Changes in QTcF during robot-assisted laparoscopic prostatectomy. Values are presented as mean ± standard deviation. QTcF: Heart-rate-corrected QT intervals calculated by Fridericia’s formula; T-5 min, 30 min, 60 min: 5, 30, and 60 min after assuming the steep Trendelenburg position with CO_2_ pneumoperitoneum; T-off: Immediately after assuming a supine position with CO_2_ desufflation. * Bonferroni-corrected *p* < 0.05 compared with the value before induction within each group.

A comparison of QT intervals over time showed no significant differences between the two groups (QT P_group×time_ = 0.5755, QTcB P_group×time_ = 0.1106, QTcF P_group×time_ = 0.4428; Figure 2, Figure 3 and Figure 4, respectively).

However, significantly increased QTc intervals, relative to those before induction, were observed 5 min after the subjects assumed the Trendelenburg position in the within-group comparison over time (QTcB and QTcF P_time_ < 0.001). The QTcF intervals in the ramosetron group 30 and 60 min after assuming the Trendelenburg position were longer than those in the control group at the same time points; however, after adjustment for multiple comparisons, no significant difference was observed between the two groups (30 min: 423 vs. 400 ms, respectively, Bonferroni-corrected *p* = 0.069; 60 min: 426 vs. 404 ms, respectively, Bonferroni-corrected *p* = 0.2454). A longer QTcB interval was observed for the ramosetron group than for the control group when subjects were returned to the supine position from the Trendelenburg position, but this difference was not significant following Bonferroni correction (443 vs. 420 ms, respectively, Bonferroni-corrected *p* = 0.1866).

These increases in the QTc interval, compared with before induction, were sustained until the end of surgery. No significant differences were observed in the number of subjects whose QTc intervals increased to greater than 450 or 500 ms between the groups (Table 2).

Increases in the QTc interval, compared with those before anesthesia, were evaluated, which revealed that more subjects experienced an increase of >40 ms in QTcF in the ramosetron group than in the control group (85% vs. 54%, respectively, *p* = 0.020). The hemodynamic and intraoperative variables were similar between the two groups (Table 3).

No significant differences in either the Tp-e interval or the Tp-e/QT ratio were observed between groups at any time point (Table 4).

## 4. Discussion

In this randomized clinical trial, ramosetron administered at 0.3 mg did not significantly increase the QTc or Tp-e intervals in subjects undergoing RALP.

The prolongation of QTc (ventricular repolarization) should be cautiously monitored during general anesthesia due to the risk of developing TdP and ventricular arrhythmia, which can cause sudden cardiac death [13]. Anesthetic agents and other perioperatively administered drugs can affect the QTc interval [6,14]. The primary mechanism through which drugs induce repolarization is the disruption of K^+^ flow through cardiac ion channels [6]. During the cardiac action potential, an inward Na^+^ and Ca^2+^ current depolarizes the cell and maintains the plateau phase, whereas an outward K^+^ current repolarizes the cell [6]. K^+^ channel blockade prolongs the action potential, resulting in the prolongation of the QTc interval [15]. 5-HT_3_ antagonists can contribute to this proarrhythmic mechanism, causing the lengthening of the repolarization period. Two important repolarizing K^+^ channels have been identified: the human ether-a-go-go-related gene (hERG) and the potassium voltage-gated channel subfamily Q member 1 (KvLQT1)/minimal potassium channel subunit (minK) [16]. The hERG channel is responsible for the rapid repolarizing current, whereas the KvLQT1/minK channel produces a slow repolarizing current. According to a previous in vitro study, ondansetron has the highest potency for blocking the hERG K^+^ channel among known 5-HT_3_ antagonists [16], and several studies have examined the effects of ondansetron on QTc prolongation [11,17,18]. However, unlike ondansetron, the effect of ramosetron on QTc prolongation remains controversial [4,11,19]. The mechanism underlying this difference in QTc prolongation between the two drugs is not well understood. Although these two drugs share some mechanisms of action, each 5-HT_3_ antagonist has a different chemical structure, which affects the receptor binding affinity, dose–response, and duration of effect. Further research is necessary to understand the action of each drug on QTc prolongation.

The QTc interval is prolonged by bradycardia and shortened by tachycardia, and the QTc interval is commonly used as a torsadogenic ECG marker. A QTc prolongation > 20 ms, relative to baseline, has been linked to an enhanced risk of TdP, and a QTc interval > 500 ms has been shown to be a strong risk factor for TdP [6]. The Tp-e interval is also a marker of ventricular arrhythmogenesis, which has been linked to sudden cardiac death [20]. Previous research suggested that ramosetron treatment increases the maximal change in the QTc interval [4], which is contrary to our findings. This difference may be associated with differences in the characteristics of the study subjects. The subjects of this previous study had severe coronary artery disease and underwent off-pump coronary artery bypass surgery [4]. Severe cardiovascular disease may increase the susceptibility of patients to the effects of ramosetron due to abnormal automaticity or impulse conditions [18].

RALP is a surgical procedure with several advantages, including reductions in blood loss and the rate of positive surgical margins, along with fewer postoperative complications and shorter duration of hospital stays [21]. RALP is performed with the subject in a steep Trendelenburg position with CO_2_ pneumoperitoneum, which causes decreases in MAP and increases in the partial pressure of arterial CO_2_ and peak inspiratory pressure. Pneumoperitoneum affects intrathoracic pressure by displacing the diaphragm upward, and the Trendelenburg position shifts the intestines and diaphragm upward, decreasing lung compliance [9]. In addition, CO_2_ pneumoperitoneum and the Trendelenburg position can cause sympathetic stimulation by increasing abdominal pressure and hypercarbia [22,23]. Sympathetic stimulation results in prolongation of the QTc interval [24]. Patients undergoing RALP tend to be older and often present with various cardiovascular diseases; therefore, prolongation of the QTc interval in these patients should be monitored cautiously. In the present study, the median prolongation of the QTc interval was >40 ms in both groups, which is relatively long when considering that a QTc prolongation of 20 ms has been associated with the development of arrhythmia [6]. However, a similar change was observed in both groups, suggesting that the administration of ramosetron (0.3 mg) did not significantly increase the QTc interval among patients undergoing RALP. Nonetheless, QTc prolongation, relative to the baseline QTc interval, was maintained after the end of the surgical procedure in both groups, with the largest QTc interval observed when returning to the supine position (T-off). These results suggested that patients who experience QTc prolongation (>500 ms) during RALP should be monitored cautiously, even after surgery [6].

This study had some limitations. First, ramosetron has a high receptor affinity and an estimated elimination half-life of 4–9 h [25]. Therefore, differences in the QTc intervals between the two groups may have persisted beyond the last time point measured in the present study. However, the highest QTc interval was observed at the T-off time during RALP in the present study, and the maximal change in the QTc interval was reported to occur 90 min after ramosetron administration in a previous study [4]. Because the median surgery time in the current study lasted >100 min, we likely captured the maximal change in the QTc interval. Further study on postoperative QTc interval is required since the QTc interval remained prolonged toward the end of the surgery. Second, this was a single-center study. Therefore, our results might not be applicable to patients who will undergo surgery in different medical settings.

The administration of ramosetron at 0.3 mg did not significantly affect the QTc or Tp-e intervals among patients undergoing RALP. Therefore, ramosetron can be safely administered to prevent postoperative nausea and vomiting among patients who are undergoing RALP.

## Figures and Tables

**Table 1:** Patient characteristics and intraoperative variables.

	Control Group (N = 28)	Ramosetron Group (N = 26)	*p*-Value
Age (years)	65.8 ± 6.5	68.0 ± 7.0	0.234
Height (cm)	168.0 ± 6.8	167.1 ± 6.5	0.628
Weight (kg)	71.1 ± 9.4	67.9 ± 9.8	0.222
ASA class (I/II)	8/20	11/15	0.441
Hypertension	15 (54)	11 (42)	0.579
Diabetes mellitus	6 (21)	3 (12)	0.543
COPD	0 (0)	2 (8)	0.439
Postoperative hospital day	2 (2–3)	3 (2–4)	0.115
Anesthesia duration (min)	160 (145–178)	160 (135–205)	0.768
Operation duration (min)	108 (95–127)	121 (103–148)	0.258
Fluid intake (mL)	1565 ± 356	1457 ± 557	0.463
Blood loss (mL)	200 (100–300)	225 (100–400)	0.732
Urine output (mL)	150 (100–240)	200 (100–200)	0.895

Values are presented as median (interquartile range), mean ± standard deviation, or number of patients (%). ASA: American Society of Anesthesiologists; COPD: Chronic obstructive pulmonary disease.

**Table 2:** Changes in QTc interval during robot-assisted laparoscopic prostatectomy.

	Control Group (N = 28)	Ramosetron Group (N = 26)	*p*-Value
QTcB interval > 450 ms	8 (29)	12 (46)	0.291
QTcB interval > 500 ms	1 (4)	2(8)	0.947
QTcF interval > 450 ms	9 (32)	9 (35)	>0.999
QTcF interval > 500 ms	1 (4)	1 (4)	>0.999
∆ QTcB interval (ms)	40.3 (26.5–62.9)	49.9 (32.4–68.4)	0.232
>20 ms	24 (86)	24 (92)	0.736
>40 ms	14 (50)	19 (73)	0.145
>60 ms	9 (32)	8 (31)	>0.999
>80 ms	2 (7)	2 (8)	0.736
∆ QTcF interval (ms)	42.0 (31.2–65.5)	49.8 (41.7–61.4)	0.253
>20 ms	26 (93)	24 (92)	>0.999
>40 ms	15 (54)	22 (85)	0.020
>60 ms	9 (32)	7 (27)	0.903
>80 ms	1 (4)	3 (12)	0.551

Values are presented as median (interquartile range) or number of patients (%). QTc: Heart-rate-corrected QT; QTcB: QT interval corrected for heart rate using Bazett’s formulae; QTcF: QT interval corrected for heart rate using Fridericia’s formulae; ∆ QTc interval: Maximal change in QTc interval.

**Table 3:** Hemodynamic profiles and intraoperative variables during robot-assisted laparoscopic prostatectomy.

	Control Group (N = 28)	Ramosetron Group (N = 26)	*p*-Value
Mean arterial pressure (mmHg)			P_group×time_ = 0.376
Before induction	98.21 ± 11.98	95.50 ± 8.40	0.3863
After induction	74.57 ± 9.20 *	78.88 ± 15.53 *	0.169
T-5 min	90.82 ± 12.90 *	94.42 ± 14.69	0.2506
T-30 min	80.59 ± 9.28 *	82.46 ± 10.97 *	0.4887
T-60 min	77.60 ± 11.83 *	81.71 ± 9.98 *	0.4667
T-off	71.43 ± 9.14 *	72.77 ± 11.96 *	0.6686
End of surgery	72.43 ± 8.57 *	77.08 ± 13.06 *	0.1384
Heart rate (beats/min)			P_group×time_ = 0.5246
Before induction	69.78 ± 10.83	71.15 ± 11.78	0.6069
After induction	68.04 ± 9.72	71.9 ± 11.22	0.1474
T-5 min	67.47 ± 8.85	66.97 ± 8.45	0.6661
T-30 min	65.86 ± 8.59	68.29 ± 9.70	0.3689
T-60 min	64.42 ± 9.38	66.61 ± 8.20	0.7098
T-off	64.64 ± 8.62 *	68.13 ± 10.15	0.1986
End of surgery	66.24 ± 11.06	67.19 ± 7.76	0.5649
EtCO_2_ (mmH_2_O)			P_group×time_ = 0.0666
After induction	35.61 ± 2.48	35.04 ± 2.66	0.725
T-5 min	42.93 ± 6.43	43.88 ± 7.77	0.5544
T-30 min	41.29 ± 6.10	43.88 ± 7.19	0.1088
T-60 min	41.12 ± 6.23	43.18 ± 6.62	0.2027
T-off	43.86 ± 5.90	47.12 ± 7.01	0.0538
End of surgery	40.11 ± 5.95	39.54 ± 4.72	0.725
EtSevo (%)			P_group×time_ = 0.6969
After induction	1.40 ± 0.24	1.35 ± 0.21	0.3609
T-5 min	1.73 ± 0.20	1.62 ± 0.27	0.0546
T-30 min	1.75 ± 0.18	1.63 ± 0.19	0.0255
T-60 min	1.74 ± 0.21	1.62 ± 0.19	0.0297
T-off	1.67 ± 0.17	1.57 ± 0.18	0.084
End of surgery	1.54 ± 0.16	1.40 ± 0.18	0.0093

Values are presented as estimated mean ± standard deviation. T-5 min, 30 min, 60 min: 5, 30, and 60 min after assuming the steep Trendelenburg position with CO_2_ pneumoperitoneum; T-off: Immediately after assuming a supine position with CO_2_ desufflation; EtCO_2_: End-tidal carbon dioxide; EtSevo: End-tidal sevoflurane concentration. * Bonferroni-corrected *p* < 0.05 compared with the value before induction within each group.

**Table 4:** Changes in Tpeak-Tend (Tp-e) interval and Tp-e/QT ratio during robot-assisted laparoscopic prostatectomy.

	Control Group (N = 28)	Ramosetron Group (N = 26)	*p* Value
Tp-e interval (ms)			P_group×time_ = 0.7503
Before induction	64.13 ± 20.86	69.74 ± 12.28	0.2394
After induction	58.59 ± 13.04	63.12 ± 9.22	0.3414
T-5 min	60.17 ± 19.92	63.82 ± 10.88	0.4555
T-30 min	61.30 ± 23.00	64.25 ± 11.72	0.8705
T-60 min	55.46 ± 12.69	63.18 ± 11.86	0.476
T-off	64.76 ± 30.25	64.45 ± 9.82	0.9393
End of surgery	61.27 ± 22.13	62.68 ± 9.66	0.7618
Tp-e/QT ratio			P_group×time_ = 0.533
Before induction	0.18 ± 0.04	0.19 ± 0.03	0.1317
After induction	0.16 ± 0.03 *	0.17 ± 0.03 *	0.2041
T-5 min	0.16 ± 0.04 *	0.16 ± 0.03 *	0.5549
T-30 min	0.16 ± 0.04 *	0.16 ± 0.02 *	0.9513
T-60 min	0.14 ± 0.02 *	0.15 ± 0.02 *	0.6552
T-off	0.16 ± 0.05 *	0.16 ± 0.02 *	0.9883
End of surgery	0.15 ± 0.05 *	0.15 ± 0.02 *	0.8215

Values are presented as estimated mean ± standard deviation. T-5 min, 30 min, 60 min: 5, 30, and 60 min after assuming the steep Trendelenburg position with CO_2_ pneumoperitoneum; T-off: Immediately after assuming a supine position with CO_2_ exsufflation. * Bonferroni-corrected *p* < 0.05 compared with the value before induction within each group.

## Data Availability

The data that support the findings of this study are available from the corresponding author on reasonable request.
